# Ontogenetic shifts in male mating preference and morph-specific polyandry in a female colour polymorphic insect

**DOI:** 10.1186/1471-2148-13-116

**Published:** 2013-06-06

**Authors:** Rosa Ana Sánchez-Guillén, Martijn Hammers, Bengt Hansson, Hans Van Gossum, Adolfo Cordero-Rivera, Dalia Ivette Galicia Mendoza, Maren Wellenreuther

**Affiliations:** 1 Departamento de Ecoloxía e Bioloxía animal, Grupo de Ecoloxía Evolutiva e da Conservación, Universidade de Vigo EUET Forestal, Campus de Pontevedra, Pontevedra 36005, Spain; 2 Behavioural Ecology and Self-organization, Centre for Ecological and Evolutionary Studies, University of Groningen, Groningen, The Netherlands; 3 Department of Biology, Ecology Building, Lund University, Lund, Sweden; 4 Evolutionary Ecology Group, University of Antwerp, Antwerp, Belgium

**Keywords:** Female limited polymorphism, Ischnura elegans, Frequency-dependent mate choice, Learned preference, Naïve males, Female polyandry

## Abstract

**Background:**

Sexual conflict over mating rates may favour the origin and maintenance of phenotypes with contrasting reproductive strategies. The damselfly *Ischnura elegans* is characterised by a female colour polymorphism that consists of one androchrome and two gynochrome female morphs. Previous studies have shown that the polymorphism is genetic and to a high extent maintained by negative frequency-dependent mating success that varies temporally and spatially. However, the role of learning in male mating preferences has received little attention. We used molecular markers to investigate differences in polyandry between female morphs. In addition, we experimentally investigated innate male mating preferences and experience-dependent shifts in male mating preferences for female morphs.

**Results:**

Field and molecular data show that androchrome females were less polyandrous than gynochrome females. Interestingly, we found that naïve males showed significantly higher sexual preferences to androchrome than to gynochrome females in experimental trials. In contrast, experienced males showed no preference for androchrome females.

**Conclusions:**

The ontogenetic change in male mate preferences occurs most likely because of learned mate recognition after experience with females, which in this case does not result in a preference for one of the morphs, but rather in the loss of an innate preference for androchrome females.

## Background

The ecological and evolutionary forces that maintain genetic polymorphisms are of interest to the evolution of alternative phenotypes. When polymorphisms are restricted to one sex this is usually explained in the context of sexual selection. Polymorphisms restricted to females, namely female-limited polymorphisms, occur in a variety of taxa including insects, fish and mammals [1,2] and are frequently observed in Odonata (damselflies and dragonflies) [3,4]. In damselflies, female-limited colour polymorphisms are common. Generally, one female morph (androchrome) resembles conspecific males in body colouration [5] and behaviour [6], whereas the other female morph(s) (gynochrome) are unlike males (or androchromes) in these traits [7].

The maintenance of female-limited colour polymorphisms in damselflies has been explained by sexual conflict over mating rates e.g. [8,9]. In promiscuous species, such as damselflies, reproductive success of males often increases with the number of matings, whereas reproductive success of females follows a law of diminishing returns, leading to a lower optimal mating frequency for females [10]. Although accurate quantifications of mating costs in wild damselfly populations are difficult to obtain, it seems likely that females suffer fitness costs from excessive male mating harassment, since sexual interactions and copulations reduce foraging time and/or increase the risks of injuries and predation [11,12]. If copulations, or mating behaviour more generally, are costly to females [13] and if one or few matings suffice for the lifetime fertilization of eggs [14], then females are expected to limit their mating frequency. Nevertheless, female damselflies usually mate several times during their reproductive lifespan, and sometimes even several times before each oviposition [15].

Investigations to understand the mechanisms governing the maintenance and evolution of the colour polymorphism in damselflies have focussed on two adaptive hypotheses; the male mimicry and the learned mate recognition hypotheses [16]. Both have in common that they assume that female polymorphism is maintained by negative frequency-dependent selection, with the primary selective force being costly male sexual harassment. They differ, however, regarding their predictions of mating harassment in response to the androchrome frequency at a population. According to the male mimicry hypothesis, androchrome females are predicted to receive less male harassment due to their resemblance to males, unless androchrome females become the majority morph in the population, at which point the mimetic protection breaks down and males should become indiscriminate between female morphs [17]. The learned mate recognition hypothesis assumes that male mating preference for a female morph increases with the encounter frequency of that female morph in the population [18]. The majority of studies to date have used field or laboratory estimates of mating rates at different morph frequencies to investigate the maintenance of female-limited polymorphisms in damselflies [19-22]. A general finding of these aforementioned studies was that androchrome females have typically lower mating rates.

Previous studies investigating morph and morph specific mating frequencies in the damselfly *Ischnura elegans* suggest that male mating harassment may promote the maintenance of this sexual mating polymorphism in females through density- and frequency-dependent processes [19-22]. In this context, male mating preferences for female morphs are of great importance and have been investigated thoroughly in mature males [19,20,23,24]. Because morph frequencies can change rapidly in populations, and female morphs not just differ in colour but also in morphology [25], fecundity and resistance to male mating attempts [11,26], it may be adaptive for males to adjust their mating preferences over time and depending on the ecological context. In addition, although there is suggestive evidence that male preference for a certain female morph depends on the previous experience with that female morph e.g. [27], the role of innate male mating preferences and ontogenetic changes in male preference have until now received little attention [28].

Here, we study mating frequencies in the wild and experimentally assess innate male preferences and learning of male preferences in *I. elegans* to investigate the extent of ontogenetic changes in male mate preferences during development. In particular, we performed male mating preference experiments using naïve and experienced mature males to investigate if innate preferences for female morphs exist and to evaluate associative learning of mate preferences. With these experiments, we can test whether male mate preferences start out as being plastic or whether males show an innate preference for a female morph type. The experiments will also allow us to evaluate the role of mate learning in this system, which has been shown to affect mating decisions in other damselfly species [29]. In addition, we use for the first time molecular markers to estimate differences in the degree of polyandry between female morphs in the wild, to get accurate measurements of mating rates of mature individuals under natural conditions that can be compared to the estimates obtained using the experimental setups. Our results indicate that *I. elegans* males lose their innate preference for androchrome females and, once they gather experience with females, either show a preference for gynochrome females or no preference for morph type. Additionally all different estimates of female mating frequencies showed that androchrome females mate less than gynochrome females.

## Results

### Proportions of first time matings of morphs

**Figure 1:**
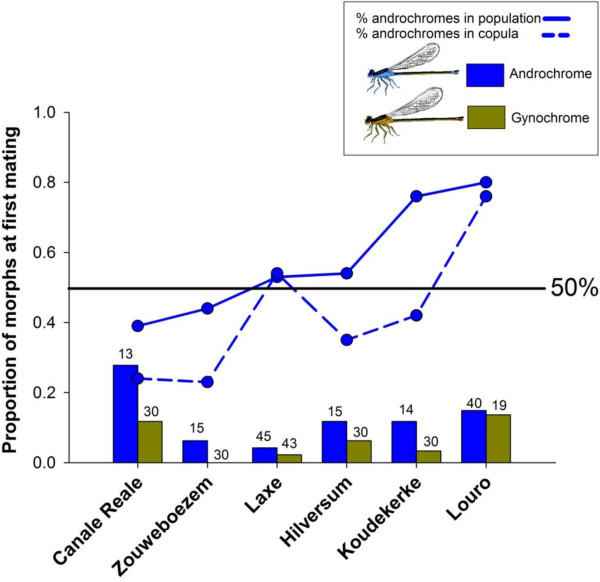
**Histogram showing the proportion of female morphs that were caught copulating for the first time, as indicated by a spermatheca without sperm.** The blue line indicates androchrome morphs. The same axis is used for all data. Androchrome frequency in the population (caught between 08:30–10:00) is denoted by a circle and androchromes in copula (caught between 10:00–15:00) by a square. The sample size number (N) of dissected androchrome and gynochrome females is indicated on each histogram.

A total of 324 females (mean per population = 54, SD ± 17.68; N = 6 populations; 143 androchrome and 181 gynochrome females) were caught in copula and dissected. Of these, 9.3% had no sperm in the spermatheca, and hence were classified as females mating for the first time. Controlling for variation in mating frequencies across populations (population was included as a factor: χ^2^ = 14.55, df = 5, p = 0.012), the proportion of androchrome females mating for the first time (19 out of 143 androchrome females, 13.3%) was higher compared to gynochrome females (11 out of 181 gynochrome females, 6.1%) (χ^2^ = 4.69, df = 1, p = 0.030; Figure 1).

### Female mating estimates from molecular markers

Twenty-eight androchrome and 34 gynochrome females from Louro and Koudekerke were dissected. Of these, four androchrome and one gynochrome females had empty spermathecae. Of the 57 females with sperm, 16 androchrome and nine gynochrome females could be successfully genotyped for at least four microsatellites. The remaining 32 sperm samples could not be genotyped due to the difficulty of extracting DNA from the sperm, dissecting sperm from the spermatheca or amplifying the small amount of DNA of each sample.

**Figure 2:**
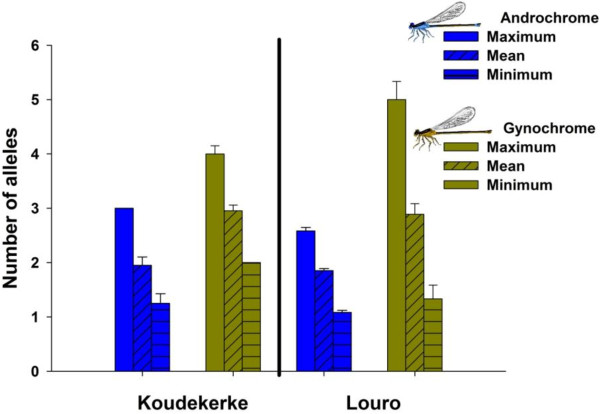
**Maximum, minimum and average estimated number of alleles (mean ± SD) for all loci.** Sample size (N) for each population is: Koudekerke androchrome females, N = 4, Koudekerke gynochrome females, N = 6, Louro androchrome females, N = 12 and Louro gynochrome females, N =3.

The average number of alleles detected in the sperm, per female across two morphs and five microsatellite loci, was 2.26 ± 0.63 (mean ± SD; N = 25, range = 1.5-3.8). Sperm collected from androchrome females contained significantly fewer alleles (1.88 ± 0.30 SD; N = 16, range = 1.5-2.5) compared to gynochrome females (2.93 ± 0.47 SD; N = 9, range = 2.5-3.8, F_1,23_ = 46.63, p < 0.001; Figure 2). In addition, androchrome females showed both a significantly lower maximum number of alleles per locus (F_1,23_ = 15.60, p < 0.001; Figure 2) and a lower minimum number of alleles (χ^2^ = 5.45, p = 0.020; Figure 2). The mean number of alleles across loci and the maximum number of alleles per locus did not differ between populations (F_1,23_ < 0.25, p > 0.624), but the minimum number of alleles detected was higher at Koudekerke (χ^2^ = 3.83, p = 0.050; Figure 2). Generally, these patterns indicate that the degree of polyandry is higher in gynochrome females.

Males from Louro (N = 15) were genotyped for the five microsatellite loci to estimate the number of male specific allele frequencies at this population. The observed heterozygosity was 0.72 and the expected heterozygosity was 0.73 while the total number of alleles was 32 and the allelic richness across loci 5.79. Based on male allele frequencies in Louro, the maximum likelihood number of mating partners, which was estimated among 1–10 mating partners, for both morphs combined was 3.38 ± 1.82 SD (N females from Louro = 17, range = 1–8). Despite the small sample size, the estimated number of mating partners was consistent with the estimated number of alleles: gynochrome females had over twice as many mating partners (5.83 ± 2.02 SD, N = 3, range = 4–8) than androchrome females (2.85 ± 1.33 SD, N = 14, range = 1–6.5). This difference between androchrome and gynochrome females was significant (Mann Whitney U Test = 2.5, N = 17, p < 0.001).

### Male sexual preferences: mature and naïve males

The sexual preference of mature males to female morphs differed between populations as models (Models 1–6, Additional file 1) containing interactions between population and female morph, and between population and presentation order, were more supported than models without these interaction terms (Additional file 1). The models containing one or both of these interactions (Models 1–6, Additional file 1) had a combined AIC weight of 0.96, whereas models without these interactions had virtually no support. Therefore we analysed sexual preferences of mature males for each population separately. In Louro and Laxe, there were no preferences for either morph type (β ± SE; Louro: -0.78 ± 0.53, Laxe: 1.87 ± 1.09), and there was no effect of presentation order (β ± SE; Louro: 0.14 ± 0.51, Laxe: -0.10 ± 0.79, Additional file 2, Figure 3). In Doniños, males preferred to mate with gynochrome females (β ± SE = 1.33 ± 0.66) and the sexual preference was higher towards the female morph that was presented first (β ± SE = 2.21 ± 0.81, Additional file 2, Figure 3).

**Figure 3:**
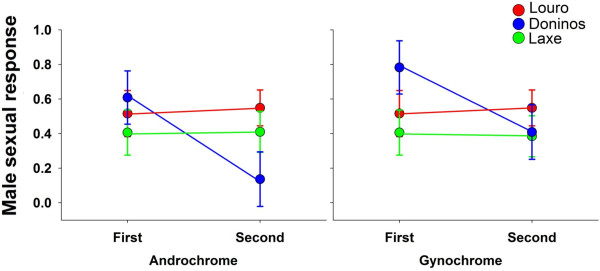
**Sexual male preferences for either androchrome or gynochrome females were tested in three populations using sequential presentations: Louro (N = 82), Laxe (N = 71) and Doniños (N = 45).** Sequential presentations (of both female morphs) were done in two orders: A-G (androchrome and gynochrome); and G-A (gynochrome and androchrome).

**Figure 4:**
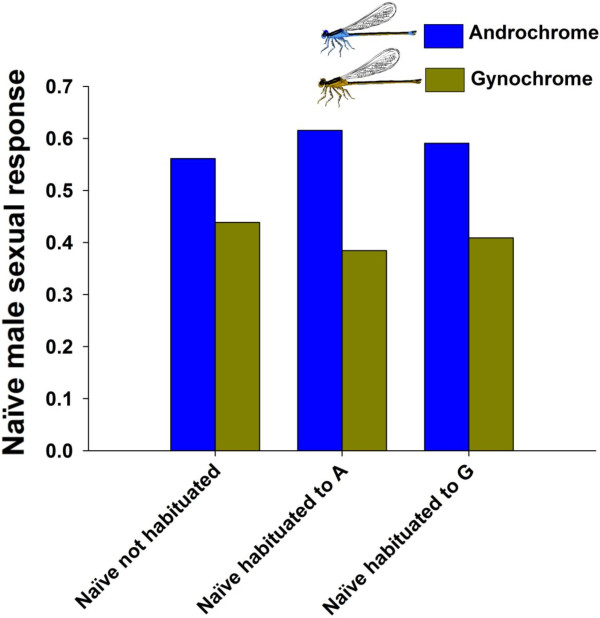
Proportion of naïve males that had no prior experience with females (Naïve), habituated to either androchrome (Naïve A) or gynochrome (Naïve G) female morphs showing sexual preference.

Unlike mature males, naïve, inexperienced males showed a higher sexual preference towards androchrome females (β ± SE = −0.63 ± 0.30, Figure 4). Interestingly, habituating naïve males to one of the two morphs did not affect male preferences as indicated by an absence of an effect of treatment type, and the absence of an interaction between treatment type and female morph (Additional file 3). Males from both treatments showed similar preferences to unhabituated males (Additional file 3, Figure 4). Although a model with an interaction between female morph and presentation was the model with the lowest AIC value in our dataset (Model 7, Additional file 3), this model was not much better than a model containing only female morph as predictor (Model 7 vs. Model 13, Additional file 3, ΔAIC = 1.15).

## Discussion

The processes governing the maintenance and evolution of heritable polymorphisms are of great interest. This is because genetic polymorphisms can be drivers of adaptive diversification through the generation of genetic and phenotypic diversity and have been implicated in the early stages of speciation [30].

Our field data showed that although morph frequencies varied widely over space and time (androchrome females ranged from 15-94% per population), morph mating frequencies were in all populations (except Laxe and Louro) lower for the androchrome females than for the gynochrome females (Table 1). Lower mating frequencies of androchrome females have previously been reported in other closely related species such as *I. ramburi*[6], *I. fluviatilis*[31], *I. graellsii*[32] and also in previous studies of *I. elegans*[11,19-21,33,34], although exceptions exists [35]. The higher mating rates in gynochrome females were consistent with field estimates of first time matings (Figure 1). We found that androchrome females were significantly more often in copula for the first time than gynochrome females, i.e. the degree of polyandry was higher in gynochrome females (Figure 1). Our field estimates of the lower mating frequencies in androchrome females were corroborated by our molecular analyses of sperm in females that were caught while copulating. Both estimates of polyandry (number of alleles, and maximum likelihood number of mating partners), were consistent with a smaller number of mating partners in androchrome females [36] Figure 2, although the estimate for maximum likelihood number of mating partners per morph type should be interpreted with caution since it is based on a small sample size of 3 individuals.

**Table 1:** Population morph and mating frequencies for the seven study populations in Spain, Italy and the Netherlands

**Locality**	**Country**	**Lat**	**Long**	**Date**	**Population frequencies**		**Mating frequencies**		**χ2-value**	***P***
					**A**	**G**	**A**	**G**		
Laxe	Spain	43.2125	−8.9554	2007 (7)	49 (152)	51 (158)	51 (217)	49 (211)	0.48	0.49
Laxe	Spain			2008 (6)	56 (157)	44 (125)	54 (373)	46 (314)	0.53	0.47
Laxe	Spain			2009 (1)	59 (26)	41 (18)	62 (51)	38 (31)	0.328	0.57
				*All (14)*	*53 (335)*	*47 (301)*	*54 (641)*	*46 (556)*	0.018	0.89
Doniños	Spain	43.2927	−8.1855	2007 (2)	21 (12)	79 (44)	5 (1)	95 (19)	3.212	0.07
Doniños	Spain			2008 (1)	37 (10)	63 (17)	13 (2)	87 (13)	3.622	0.06
Doniños	Spain			2009 (3)	15 (19)	85 (107)	7 (7)	93 (91)	4.823	***0.03***
				*All (6)*	*20 (41)*	*80 (168)*	*8 (10)*	*92 (123)*	12.344	***<0.001***
Louro	Spain	42.7580	−9.0953	2007 (5)	78 (236)	22 (65)	79 (58)	21 (15)	0.047	0.83
Louro	Spain			2008 (6)	76 (158)	24 (49)	74 (29)	26 (10)	0.084	0.77
Louro	Spain			2009 (2)	94 (98)	6 (6)	74 (78)	26 (27)	76.789	***<0.0001***
				*All (13)*	*80 (492)*	*20 (120)*	*76 (165)*	*24 (52)*	2.611	0.1061
Canale Reale	Italy	40.4200	17.490	2007 (1)	39 (44)	61 (68)	24 (13)	76 (41)	5.234	***0.0221***
Hilversum [21]	Netherlands	52.1326	5.1015	2007 (1)	54 (37)	46 (32)	35 (14)	65 (26)	5.58	***0.0182***
Koudekerke [21]	Netherlands	51.2909	3.3225	2007 (1)	76 (25)	24 (8)	42 (36)	58 (49)	51.624	***<0.0001***
Zouweboezem [21]	Netherlands	51.5705	4.5955	2007 (1)	44 (19)	56 (24)	23 (12)	77 (40)	9.401	***0.0022***
All					*993*	*721*	*891*	*887*	*44.641*	***<0.0001***

Observed and expected mating frequencies were compared with a χ2-test. A denotes androchrome and G gynochrome females. Morph percentages are given before and morph numbers (N) within brackets.

Though the field and molecular data on mating frequencies were consistent, we noted that the molecular data yielded more sensitive measures on mating frequencies, allowing the detection of smaller differences in mating numbers than classical field observations. For example, it was not possible to find significant differences between the observed and expected mating frequencies in the androchrome females from Louro (pooled across years) when we only based our analyses on morph and mating frequencies (Table 1). However, when we estimated the female mating frequencies using sperm genotyping, we found that the androchrome females from Louro mated less than expected based on the morph frequencies. This observation might be important when one wants to investigate the effects of small mating differences between populations, and in such a case, we suggest that molecular methods should be employed. It should also be noted, however, that molecular methods have limitations. First, females have to be collected and often killed to estimate their copulation histories, and this means that we can only study part of their life. Second, if males that have mated with the same female share one or more alleles, then it is not possible to detect all possible partners, and the evaluation of the minimum number of matings will be underestimated (yielding a conservative estimate). Third, the dissection of genitalia and the application of molecular methods also contain some methodological difficulties (only half of our samples could be successfully genotyped) and these methods imply additional costs (e.g. chemicals and labor).

We tested male sexual preferences for female morphs in populations with contrasting androchrome frequencies (20, 53 and 80% androchrome females, Table 1). Using sequential presentation trials, we detected either preference for gynochrome females (in Doniños and Laxe) or no preference (in Louro) in experienced males (Figure 3). The lack of a clear morph preference of experienced *I. elegans* males in our experiments, in combination with the lower mating frequencies of androchrome females in natural populations suggests that male–female mating interactions, significantly affect the outcome of male mating attempts. Moreover, this outcome is morph-specific and irrespective of the population morph frequency. We will discuss the two most likely factors affecting the outcome of male mating attempts below.

In damselflies, sperm from one mating is typically sufficient to fertilize all eggs of a single female. However, most damselfly females are involved in multiple matings. This is partly explained by the benefits a female may receive by mating more than once, for example, by obtaining compatible or superior sperm from multiple matings, which results in an increase in the fitness of their offspring [37]. However, if female morphs differ in the amount of reproductive reserves, such as in *Ischnura denticollis* and *Enallagma novahispaniae* see [38], then the costs of multiple matings would be higher in the morph with the lower resources. Previous work has shown that androchrome females are less fecund than *infuscans* females [22,39] and this suggest that androchrome females would gain less from accepting multiple matings and consequently might be less willing to engage in additional mating interactions after they have mated once. Support for this comes from behavioural studies showing that when androchrome females are approached by a male, they respond more aggressively towards mating attempts than gynochrome females [6,40] and are more reluctant to engage in multiple matings [41]. Lower mating rates of androchrome females may also be caused by androchrome behaviour that, in addition to colour and morphology, mimics males thereby making it difficult for males to distinguish between males and androchrome females (mimicry), or because they show aggressive behaviour towards approaching males. This idea is supported by studies showing that androchrome females perch at similar heights as males, spend less time than gynochrome females hidden in the vegetation and fly less than gynochrome females [40]. This strongly suggests that in addition to colour and morphology imitation, behaviour imitation plays a crucial role in the maintenance of this colour polymorphism as has been previously suggested [33,42].

In this sense, the preference for gynochrome females (2 populations) or the lack of a morph preference (1 population) in mature males together with the androchrome specific characteristics most likely explains the lower androchrome mating rates in the wild. In addition to our field and molecular data on mature male–female mating interactions, we used, for the first time in *I. elegans*, experiments on naïve males to assess innate male sexual preferences for female morphs. In contrast to mature male preferences, we found that naïve males showed a clear preference for androchrome females and this result was independent of treatment type (Figure 4), suggesting no role for associative learning, but rather an innate preference for androchrome females. An innate preference for a particular female morph may indicate that this preference is ancestral, which is supported by molecular phylogenetic work on *Ischnura* and *Enallagma* species indicating that female polymorphism has evolved multiple times, and that there is weak evidence that male mimics and/or blue coloration are ancestral in these groups [4].

Our study shows that males undergo an ontogenetic shift in their preference for female morphs. Males start out by showing an innate preference for androchrome morphs and over time loose this preference, i.e. mature males either show a lack of a preference or a preference for gynochrome females (Figure 3). Our results add to the emerging view that learning of mate preferences is widespread in nature, occurring in most animal taxa [43]. Studies on learning in damselflies are just emerging, but they show that damselflies have the ability to learn rapidly and quickly, and that this can be highly dependent on the ecological context as shown in *Calopteryx* spp. e.g. [29]. In another recent study by Takahashi and Watanabe [28], males of *I. senegalensis* were shown to change their mating preference for female colour depending on previous copulation experiences with sexually mature females, indicating the ability of males to learn mate preferences based on sexual history. Our result of an innate male preference of androchrome females and the lack of a habituation effect on male mate preferences contrasts with studies on related species. Studies using males of *Enallagma civile* and *I. senegalensis* reared in isolation from females did not show any preferences towards female colour morphs [28,44], and males of *E. civile* also showed no associative learning after habituation to one of the morphs [44]. The lack of a short term effect of the habituation treatment might indicate that learning in this species takes longer time than the duration of the experiment in our study. In some species of damselflies, mate preferences to discriminate against heterospecific mates are only learned following courtship interactions with the other species [29] and in colour polymorphic damselflies, males may only learn to prefer the morph with which they had a successful mating experience [44]. The number that is needed of these interactions so that a detectable change in preferences can be detected is not known but our study suggest that it is likely higher than what can be achieved during 48 hours [28]. Finally, we would like to stress that experiments using naïve individuals to estimate the genetic component of mate preferences have been underutilised in *I. elegans* and other odonate species despite such experiments offering tremendous potential to gain insights into the dynamic nature of mate preferences. We hope that more studies will evaluate naïve mate preferences in the future, particularly in phylogenetically related polymorphic species, to facilitate analyses of broader patterns, for example, the cross-species extent of innate male mate preferences and the stability of those over time. Such insights will provide valuable clues regarding the processes underlying male mating harassment in insect like *I. elegans*, and thereby ultimately also into the processes that maintain the polymorphism in populations over space and time.

## Conclusion

Taken together, our results indicate that *I. elegans* males lose their innate preference for androchrome females and, once they mature and gather experience with females, either show a preference for gynochrome females or no preference for morph type. Furthermore, the field estimates and molecular analysis showed that androchrome females mate less than gynochrome females, presumably because androchrome females mimic males in morphology and behaviour and because they are more aggressive towards males. The ontogenetic change in male mate preferences occurs most likely because of learned mate recognition, which in this case does not result in a preference for one of the morphs, but rather a loss of an innate preference for androchrome females. The importance of male mate choice in insects has received significant support over the last years [45-47] and learning in this context can be an important component of mate preference formation, where preferences are often influenced by the phenotypic variation that individuals encounter throughout their lives [43]. The modification of male morph preferences observed in our study has parallels to that observed in invertebrate predators that learn to avoid certain colours of aposematic prey [48,49]. Learned mate discrimination depending on copulation experience might help males to detect potential mates more effectively and to avoid sexually unreceptive females.

## Methods

We estimated female mating frequencies by using three different methods: 1) in seven populations, we estimated female colour morph frequencies and mating frequencies in the field; 2) all females captured on mating were dissected in order to detect if they were in the first or successive matings; and 3) in two out of the seven populations, we additionally estimated female mating frequencies by genotyping the sperm stored in the female receptacle. Furthermore, innate and learned male preference was investigated in three natural populations and in the laboratory using mature, naïve (males without experience) and naïve habituated males to one female morph in the female-limited polymorphic damselfly *Ischnura elegans*.

### Study species

*Ischnura elegans* is among the most common damselfly species in Europe [50,51]. The species is best known for its heritable female-limited colour polymorphism [7]. Mature females occur as one of three discrete morphs: one blue, male-like, androchrome morph and two green-brown gynochrome morphs, which are called *infuscans* and *infuscans-obsoleta*[33]. Although the colouration of androchrome females and conspecific males is virtually identical, they can be distinguished by visual examination of external genitalia and abdomen width [5]. The polymorphism is controlled by a single autosomal locus with three alleles under a simple dominance hierarchy: androchrome > *infuscans* > *infuscans-obsoleta*[7]. *Ischnura elegans* is a non-territorial species without male courtship [52]. Males choose females for matings by grasping the female by their prothorax (tandem position), and them the female must flex dorsally her abdomen resulting in the contact of the mating organs (wheel position) [53]. *I. elegans* females have two discrete organs of sperm storage: a single spermatheca and a single bursa copulatrix [54]. Copulation activity can be divided into three different behavioural phases corresponding to different internal genitalic activity [55]. The first stage consists on sperm removal from previous matings [56]. *I. elegans* males can remove sperm from the bursa but appear to have no access to the sperm stored in the spermatheca [52,54]. Likely, sperm stored in the spermatheca is for long-term usage [57]. The second stage consists on the insemination, sperm is initially stored in the bursa, but is later transferred to and stored in the spermatheca, where sperm mixing from previous matings may occur. The third stage is the mate guarding, in order to avoid that the female mate again before eggs oviposition, take place in the wheel position [58]. Mating in this species begins early in the morning and has one of the longest copulation durations among odonates (sometimes more than 7 h) [52]. In the family Coenagrionidae, the amount of sperm transferred from a single mating appears sufficient to fertilize a female’s lifetime number of eggs [41,59]. Nevertheless, females often mate with multiple males during their life [60].

### Field morph and mating frequencies

Observations of morph mating frequencies were done between 2007 and 2009 at seven populations in Spain (Laxe, Louro and Doniños), Italy (Canale Reale) and the Netherlands (Hilversum, Koudekerke and Zouweboezem). Data of female morph frequencies for the two gynochrome females were pooled because males show similar preferences for the gynochrome morphs [24] and because *infuscans-obsoleta* is virtually absent from North-West Spain [7,9]. Populations were only sampled on calm and sunny days with temperatures above 20°C, since this is when damselflies show maximum reproductive behaviour. At each population, damselflies were caught between 08:30–10:00, when most individuals were still unmated, by sweeping an insect net randomly through all types of vegetation within 10 m of the water. The sex, age (mature or immature) and morph (androchrome or gynochrome) of all captured individuals was recorded. Mature males and females are unambiguously identified by its thorax colouration see [61], and mature females additionally by their wider abdomen [7]. Morph frequencies were estimated as the number of each mature female morph divided by the total number of mature females (Table 1). During the peak period of reproductive activity between 10:00–15:00 hours, mating pairs were also caught at each population, only one sampling by population (in 2007), except for Louro where three samplings in consecutive days were necessary because of the low gynochrome frequencies. Additionally, in order to avoid that the more frequent morph were sampled earlier in the morning than the lower frequent female morph, we sampled one androchrome female and one gynochrome female each time. Mating frequencies were the number of each mature morph in copula divided by the total number of mature morphs in copula (Table 1). For each population (except Canale Reale), 43–88 mating females were caught and stored in ethanol for subsequent sperm analyses and DNA extraction. All males and unmated females were marked prior to release to avoid multiple counts of the same individual.

### Frequencies of first time matings of morphs

Three hundred and twenty four females (143 androchrome and 181 gynochrome females) captured in copula in 2007 (Table 1) from all populations (except Canale Reale) were dissected under a binocular microscope (BX40, Olympus) to dissect the bursa and spermatheca. All females without sperm in the spermatheca had mated at least once (they were collected while mating), and thus were classified as females in the first mating, while females with sperm were classified as females in second or subsequent matings. This is because all females were captured in copula with a male, though the sperm of this male may have not arrived in the spermatheca yet. Bursa and spermatheca with sperm were preserved for DNA extraction. The presence/absence of sperm in the spermatheca was analysed in a generalized linear model with a binomial distribution and a logit link function. The significance of parameters was determined from the- Wald χ^2^ statistic. Female morph (androchrome or gynochrome) and population were categorical predictors. Interactions between female morph and population were tested but are only reported when statistically significant.

### Morph mating frequencies using molecular markers

The sperm from the spermatheca of 28 androchrome and 34 gynochrome female morphs from Louro and Koudekerke was extracted to assess differences in number of partners between morphs (androchrome and gynochrome). For this, previously dissected spermathecae were transferred to a drop of water, placed on a slide, opened and then the sperm mass dissected by means of a micropipette (Eppendorf). The mixture of water and sperm was placed in a 1.5 ml Eppendorf tube and incubated for 40 hours at 40°C, with the occasional agitation in 0.2 ml of extraction buffer [Tris HCL (pH = 8; 0.5 M), EDTA (pH = 8; 0.5 M), NaCl (5 M), Dithiothreitol DTT (1 M) and Sodium Lauryl Sulfate SDS (10%; pH = 7.2), 3 μl of RNAse (10 mg/μl) and 5 μl of proteinase K (10 mg/μl)]; and then extracted with phenol-chloroform (1:1) before EtOH precipitation (EtOH 100% and NH_4_Ac, 4.4 M) for 24 hours at 4°C. DNA sperm was extracted using a phenol/chloroform–isoamylalcohol protocol [62] before EtOH precipitation. Pellets were eluted in 40 μl of 1 × TE (Applichem) and the concentration was estimated by comparing the intensity of bands on 1% agarose gels to a known amount of DNA standard. DNA samples were stored in 1 × TE at a standardized concentration of 0.25-2.5 ng/μl. Genomic DNA was genotyped using five microsatellite loci [63] in 10 μl reactions: 1–5 ng genomic DNA, 1 unit of Platinum-taq polymerase (Invitrogen), 15 nmol of MgCl_2_, 1.25 nmol of dNTP mix and 4 pmol of each primer (Metabion). PCR amplifications included an initial denaturation step of 94°C for 2 min, followed by 35 cycles of 94°C for 30 s, touch-down from 62–58°C for 30 s, 72°C for 30 s and a final extension step of 72°C for 10 min. Multiplex primer reactions were performed for combinations of primers with matching annealing temperatures but differing size ranges and dye labels. The PCR products were then mixed with a labelled size standard and electrophoresis was conducted on an ABI-PRISM 3730 Genetic Analyzer and analyzed with GeneMapper for allelic designations.

When sperm was genotyped, we estimate maximum, minimum and average number of alleles (mean ± SD) for all loci, per female morphs. The difference in maximum and mean number of alleles per female morphs were analysed in a general linear model. The minimum number of alleles (1 or 2 alleles in all cases), was analysed in a generalized linear model with a binomial distribution and a logit link function. The significance of parameters was determined from the Wald χ^2^ statistic. Female morph (androchrome or gynochrome) and population were categorical predictors. Interactions between female morph and population were tested but are only reported when statistically significant.

This method is, however, likely to underestimate the number of matings since it does not take allelic population frequencies, homozygous loci, multiple matings or shared alleles into account. To partly address these shortcomings, we used the method developed by Bretman & Tregenza [36] which estimate the maximum likelihood number of mating partners from population allele frequencies. To estimate the maximum likelihood number of mating partners of females from Louro (N = 17), the DNA from the muscle thorax of 15 males was extracted and genotyped for the six microsatellites, following the protocol explained in previous section. Allele frequencies for each locus were estimated using the program FSTAT [64]. We used the formula: *P* (observed) = 1 − *P* (not observed), and *P* (not observed) = [1 − *f* (*a*)] *t*, where, *f*(*a*) denotes the allele frequency and *t* the number of attempts at observing it, which is twice the number of males contributing it [36]. This method calculates the probability of observing certain allelic frequencies for a determinate number mating partners, thus the number of trials with the highest probability will indicate the most likely number of males contributing to the array. When using more than one gene, the maximum-likelihood number of partners for each female is estimated using the locus showing the highest degree of putative partners. The difference in the maximum likelihood number of mating partners between female morphs was analysed using a Mann Whitney U test.

### Male sexual preferences: mature and naïve males

Mature males and females were caught at three populations with contrasting androchrome frequencies (Louro: 80%, Laxe: 53% and Doniños: 20%) to test for differences in male sexual preferences between populations and morphs and to test for an effect of the morph presentation order. Two types of sexual experiments were carried out to test male preferences for female morphs: The first experiment uses mature, experienced males that already have experience with females in the focal population, i.e. their mating preference is the outcome of innate preferences and learning. The second experiment uses naïve and inexperienced males (see further on how these were obtained) to investigate the innate preferences and the effect of association learning.

In the first experiment, the sexual preferences were recorded in the following way: one mature, experienced female (androchrome or gynochrome) was introduced to a 50×50×50 cm insectary after which a focal male was introduced, by holding the male by the wings to ensure the male was in visual contact with the female before release (n = 396). The male and female were then allowed to interact for a maximum of 2 mins [40]. During each trial, male mate preferences were tested by sequential presentations of an androchrome and a gynochrome female to each male, at a random order. Male responses were classified in two categories: 0 “no sexual response” when the male either did not respond at all or simply moved towards the female without contact, and 1 “sexual response” when the male tried to grasp the female to form a tandem [19,44]. All presentations were conducted at the population of origin to minimize variability in environmental conditions.

For the second experiment, the sexual preferences of naïve males from Louro (n = 182) were tested using three different treatments: unmanipulated naïve males, naïve males habituated to androchrome females and naïve males habituated to gynochrome females. To obtain naïve *I. elegans*, last instar larvae were caught in excess and reared following standard methodology [7]. Larvae started to emerge after 2–3 weeks and kept in individual jars that were shielded from each other and fed *Drosophila melanogaster*. Males reached sexual maturity on day 6. Emerging females were maintained in 50×50×50 cm insectaries [7], with a maximum of 15 females per insectary, and used in the preference trials once they reached sexual maturity on day 8. Preference trials were conducted as explained for the first experiment above but with naïve, unmanipulated males or naïve, habituated males. Habituation treatments prior to the trials lasted for 48 hours, during which the males and females could freely interact. During the time of habituation, males were allowed to have sexual interactions with the correspondent females.

Data from the two experiments were analysed using generalized linear models with a binomial distribution and a logit link function. The dependent variable was the male mate response variable (sexual response = 1 or no sexual response = 0). The predictor variables were: Population (experiment 1 only), female morph (androchrome/gynochrome), presentation order, treatment type (unmanipulated naïve males, naïve males habituated to androchrome females and naïve males habituated to gynochrome females) (experiment 2 only) and all two-way interactions between independent variables. The most parsimonious model was selected using Akaike’s Information Criterion (AIC). In addition, we calculated the likelihood of a focal model using AIC weights. For models that gained similar support (ΔAIC < 2), we selected the model with the fewest number of parameters as the most parsimonious model [65].

### Ethical approval

All observations and experiments complied with the current laws and ethical guidelines for Spain, The Netherlands and Italy. Permits to capture damselflies in Spain were issued by each Regional Government to RSG.

## Competing interests

The authors declare that they have no competing interests.

## Authors’ contribution

Conceived and designed the study: RAS-G, MH, BH, HVG, AC-R and MW. Performed the experiments: RAS-G, MH, DIG-M. Analyzed the data: RAS-G, MH, MW. Contributed materials/analysis tools: RAS-G, MH, BH, HVG, AC-R. Wrote the first draft of the paper: RAS-G, MW. Contributed to the final draft of the paper: RAS-G, MH, BH, HVG, AC-R, MW. All authors read and approved the final manuscript.

## Supplementary Material

Additional file 1 **Summary table of model selection statistics (AIC values) of the effects of population (P), female morph (M) and presentation order (O) on male sexual responses towards females.** The selected model is indicated in bold. Click here for file

Additional file 2 **Summary table of model selection statistics (AIC values) of the effects of female morph (M) and presentation order (O) on mature male sexual responses towards females for the three populations separately.** The model with the lowest AIC value is indicated in bold and the selected, most parsimonious model is indicated in italic. Click here for file

Additional file 3 **Summary table of model selection statistics (AIC values) of the effects of habituation treatment (T), female morph (M) and presentation order (O) on male sexual responses towards females.** The model with the lowest AIC value is indicated in bold and the selected, most parsimonious model is indicated in italic. Click here for file
