# Supplementary material for: Ontogenetic shifts in male mating preference and morph-specific polyandry in a female colour polymorphic insect
Source: BMC Evol Biol. 2013 Jun 6;13:116. doi: 10.1186/1471-2148-13-116 (PMC3691580; doi:10.1186/1471-2148-13-116)
Supplement: Additional file 1 — Summary table of model selection statistics (AIC values) of the effects of population (P), female morph (M) and presentation order (O) on male sexual responses towards females. The selected model is indicated in bold. [file 1471-2148-13-116-S1.doc]

### STable 1. Summary of model selection statistics (AIC values) of the effects of population (P), female morph (M) and presentation order (O) on male sexual responses towards females. The selected model is indicated in bold.

|  |  |  |  |  |  |  |  |
| --- | --- | --- | --- | --- | --- | --- | --- |
| **Model number** | **Model** | | | | **AIC** | **ΔAIC** | **AICWeight** |
| 1 | P + M + O + (P x M) + (P x O) + (M x O) | | | | 248.06 | 0.76 | 0.29 |
| 2 | P + M + O + (P x M) + (M x O) | | |  | 250.14 | 2.85 | 0.10 |
| 3 | **P + M + O + (P x M) + (P x O)** | | |  | **247.30** | **0.00** | **0.42** |
| 4 | P + M + O + (P x O) + (M x O) | | |  | 252.58 | 5.28 | 0.03 |
| 5 | P + M + O + (P x M) | |  |  | 250.03 | 2.74 | 0.11 |
| 6 | P + M + O + (P x O) | |  |  | 253.17 | 5.88 | 0.02 |
| 7 | P + M + O + (M x O) | |  |  | 255.12 | 7.83 | 0.01 |
| 8 | P + M + O |  |  |  | 255.58 | 8.29 | 0.01 |
| 9 | P + M |  |  |  | 257.35 | 10.05 | 0.00 |
| 10 | P + O |  |  |  | 254.62 | 7.32 | 0.01 |
| 11 | M + O |  |  |  | 260.15 | 12.85 | 0.00 |
| 12 | P |  |  |  | 256.38 | 9.08 | 0.00 |
| 13 | M |  |  |  | 262.21 | 14.92 | 0.00 |
| 14 | O |  |  |  | 259.16 | 11.87 | 0.00 |
| 15 | - |  |  |  | 261.22 | 13.92 | 0.00 |
|  |  |  |  |  |  |  |  |
