# Supplementary material for: Ontogenetic shifts in male mating preference and morph-specific polyandry in a female colour polymorphic insect
Source: BMC Evol Biol. 2013 Jun 6;13:116. doi: 10.1186/1471-2148-13-116 (PMC3691580; doi:10.1186/1471-2148-13-116)
Supplement: Additional file 2 — Summary table of model selection statistics (AIC values) of the effects of female morph (M) and presentation order (O) on mature male sexual responses towards females for the three populations separately. The model with the lowest AIC value is indicated in bold and the selected, most parsimonious model is indicated in italic. [file 1471-2148-13-116-S2.doc]

### STable 2**.** Summary of model selection statistics (AIC values) of the effects of female morph (M) and presentation order (O) on mature male sexual responses towards females for the three populations separately. The model with the lowest AIC value is indicated in bold and the selected, most parsimonious model is indicated in italic.

|  |  |  |  |  |
| --- | --- | --- | --- | --- |
|  |  | **Louro** | **Laxe** | **Doniños** |
| Intercept only | | *115.49* | 57.79 | 83.10 |
| M |  | **115.20** | ***55.64*** | 81.05 |
| O |  | 117.42 | 59.77 | 75.05 |
| M + O |  | 117.13 | 57.63 | ***72.54*** |
| M + O + (M x O) | | 117.14 | 58.37 | 73.45 |
